# Recent genetic advances on boar taint reduction as an alternative to castration: a review

**DOI:** 10.1007/s13353-020-00598-w

**Published:** 2021-01-06

**Authors:** Darlene Ana Souza Duarte, Martine Schroyen, Rodrigo Reis Mota, Sylvie Vanderick, Nicolas Gengler

**Affiliations:** TERRA Teaching and Research Centre, Gembloux Agro-Bio Tech, University of Liège, 5030 Gembloux, Belgium

**Keywords:** Androstenone, Skatole, Castration alternatives, Genetic selection, Pigs

## Abstract

Boar taint is an unpleasant odor in male pig meat, mainly caused by androstenone, skatole, and indole, which are deposited in the fat tissue. Piglet castration is the most common practice to prevent boar taint. However, castration is likely to be banished in a few years due to animal welfare concerns. Alternatives to castration, such as genetic selection, have been assessed. Androstenone and skatole have moderate to high heritability, which makes it feasible to select against these compounds. This review presents the latest results obtained on genetic selection against boar taint, on correlation with other traits, on differences in breeds, and on candidate genes related to boar taint. QTLs for androstenone and skatole have been reported mainly on chromosomes 6, 7, and 14. These chromosomes were reported to contain genes responsible for synthesis and degradation of androstenone and skatole. A myriad of work has been done to find markers or genes that can be used to select animals with lower boar taint. The selection against boar taint could decrease performance of some reproduction traits. However, a favorable response on production traits has been observed by selecting against boar taint. Selection results have shown that it is possible to reduce boar taint in few generations. In addition, modifications in diet and environment conditions could be associated with genetic selection to reduce boar taint. Nevertheless, costs to measure and select against boar taint should be rewarded with incentives from the market; otherwise, it would be difficult to implement genetic selection.

## Introduction

Boar taint is an unpleasant odor in male pork meat caused mainly by the deposition of androstenone, skatole, and indole in fat tissue. Boar taint could be perceived as a urine or fecal-like odor. Moreover, the odor was described as sweat, manure, and naphthalene (Dijksterhuis et al. 2000). The sensitivity of consumers may vary, with some consumers not being able to detect the odor (Weiler et al. 2000).

Androstenone is synthesized in the testes along with other steroid hormones, and it degrades in the testes and liver. Skatole and indole are produced in the intestine, starting from tryptophan, and it is degraded in the liver. Androstenone, skatole, and indole are deposited in fat tissue at sexual maturity (Babol et al. 2004; Zamaratskaia et al. 2004b).

The most common practice to prevent boar taint is piglet castration at an early age. However, this practice is not in accordance with animal welfare, and it has been under social pressure. For these reasons, an agreement by the European Commission stated that surgical castration should be eliminated by 2018. Feasible alternatives have been studied but are not fully implemented.

Immunocastration, slaughter at young age, gender selection, and altering the management system may be considered piglet castration replacements (Lundström and Zamaratskaia 2006; Valeeva et al. 2009). There are concerns regarding immunocastration acceptance by consumers, mainly due to possible long-term side effects of vaccines. More studies regarding consumers’ acceptance are still required, since only a small proportion of consumers have knowledge about immunocastration, as discussed by Mancini et al. (2017). Slaughter at young age and gender selection do not appear to be a profitable alternative. Some adaptations to the current management system such as an extra clean environment, especially just before the slaughter, decreasing the number of animals per pen, keeping the same social groups, and others have been suggested (Hansen et al. 1994; Aldal et al. 2005; Van Wagenberg et al. 2013).

Valeeva et al. (2010) showed that genetic selection is a cost-effective alternative, although the authors have proposed that more than one alternative to piglet castration should be adopted to ensure boar taint-free pig meat production. Heritabilities for androstenone and skatole are from medium to high magnitudes (Sellier and Bonneau 1988; Tajet et al. 2006; Parois et al. 2015). The challenge here concerns their correlations with other traits, in particular reproduction and meat quality. In this review, we aimed to discuss recent genetic advances in boar taint reduction focusing on (1) the correlation between economically important traits in pigs and boar taint traits; (2) the indirect response to selection against boar taint; and (3) potential candidate gene findings via association studies. Additional considerations will be given to phenotyping strategies, optimum use of relatives, microbiome × host genome interactions, and recent alternatives based on the use of gene editing tools, such as CRISPR/Cas.

## Boar taint compounds

The principal compounds found to be causative of boar taint are androstenone, skatole, and indole. Androstenone (5α-androst-16-ene-3-one) is a steroid formed in the Leydig cells of the testes and its synthesis is regulated by the luteinizing hormone (LH) (Robic et al. 2008). This compound is normally degraded in the liver and testes, and it is eliminated through the urine. On the other hand, the non-degraded androstenone is deposited in fat tissue (Robic et al. 2008).

Skatole (3-methylindole) causes fecal-like odor in male pork meat, and consumers may be highly sensitive to it. Skatole is a product of tryptophan breakdown by bacteria in the intestinal colon and its function in pigs remains unknown. In the same way as androstenone, the remaining non-degraded skatole accumulates in the fat tissue (Andresen 2006). Skatole seems to contribute more than androstenone to odor perception, whereas both contribute similarly to flavor (Matthews et al. 2000; Whittington et al. 2011). Moreover, there are people insensible to androstenone odor (Weiler et al. 2000).

As skatole, indole (2,3-benopyrol) is produced in the intestine from the breakdown of tryptophan. Indole seems to accentuate the feces odor provoked by skatole but is not the main responsible.

Other substances have been identified as contributing to the off odor in the meat such as aldehydes, short-chain fatty acids (Rius et al. 2005), and 4-phenyl-3-buten-2-one (Rius Solé and García-Regueiro 2001). These compounds could attenuate the perception of androstenone and skatole, even at low levels. Skatole levels tend to increase in fat tissue in non-castrated males during puberty (Babol et al. 2004; Zamaratskaia et al. 2004b); this could happen due to the increase of steroid hormones (Doran et al. 2002; Zamaratskaia et al. 2004a). Doran et al. (2002) demonstrated that androstenone blocks the induction of *CYP2E1* by skatole, resulting in a reduced skatole metabolism and its accumulation in adipose tissue. Moreover, *CYP2A6* (known as *CYP2A19* cytochrome P450 2A19*)* has also a role in the degradation of skatole (Diaz and Squires 2000), and its expression is inhibited by androstenone, while it is stimulated by skatole and indole (Chen et al. 2008).

## Methods for measuring boar taint

In order to make genetic selection possible, relevant phenotypes are necessary. Measuring boar taint can be a very tricky task considering that there is a wide range of methods to assess boar taint and they can bring different outcomes. These different types of phenotypes shape also possibilities for genetic selection and are influenced by several non-genetic factors. Therefore, a brief discussion on those methods is given in this section, with special consideration for breeding related issues.

In general, the methods to asses boar taint can be divided into two large groups: analytical measures of compound concentration (or related substances), or direct scoring methods.

Assessments can be done on the slaughter line, mostly by direct scoring methods, but also by automatized methods (e.g., electronic nose) that are under development in the food industry (Loutfi et al. 2015). Samples collected in slaughterhouses can be either taken to the laboratory for analysis or brought to a panel of specialists to be evaluated (Aluwé et al. 2011a; Haugen et al. 2012). The samples are often collected from the backfat of the neck region (Haugen et al. 2012; Mathur et al. 2012; Bekaert et al. 2013).

Although most samples are collected after slaughter, it should be highlighted here that some phenotyping methods can be adapted to be used on living animals (e.g., by biopsy, urine, and blood) (Baes et al. 2013; Wauters et al. 2015; Jacob et al. 2017). Therefore, they are potentially usable on close relatives of animals to be slaughtered (e.g., sire). This would provide earlier information on a breeding animal and enable access to its own performance, being of great interest for candidate animals for selection. Information on closely related animals, as full-brothers that are not kept for reproduction, can be also added to the pool of data.

Generally, using chemical analysis, concentration of androstenone, skatole, and indole can be analyzed using different methods. These analyses are laborious and not appropriate to be used directly in slaughter lines and therefore less adapted for routine assessments as necessary for genetic evaluations. There are numerous analytical methods used to evaluate these compounds; a review of those methods can be found in Haugen et al. (2012), where the authors highlight the need for a standardization of methods to quantify boar taint compounds. Additionally, new methods that can be used on the slaughter lines were reviewed by Font-i-Furnols et al. (2020).

Also, other compounds have been considered. Recently, plasma estradiol has been suggested as a predictor of androstenone (Prunier et al. 2016). High genetic correlations have been found between androstenone in fat tissue and estradiol in plasma (ranging from 0.8 to 0.96) (Grindflek et al. 2011; Dugué et al. 2020). Thus, plasma estradiol, taken on living animals, could be used to predict androstenone in fat tissue and, therefore, to select animals with low levels of androstenone.

The sensory analysis performed by experts or consumers, selected according to their ability to perceive androstenone and skatole, classifies samples according to the level of odor (Bekaert et al. 2013; Trautmann et al. 2016). Different aspects of sensory analysis were discussed in Font-i-Furnols (2012). A variant of this approach is the human nose scoring (HNS), which can be performed by an expert (panel) in the slaughter line. Due to this fact, HNS has been considered the easiest, fastest, and less costly detection method (Aluwé et al. 2012; Mathur et al. 2012) and well adapted for routine assessments inside a genetic evaluation system. This method has presented moderate to high phenotypic and genetic correlations with androstenone and skatole concentration (Whittington et al. 2011; Mathur et al. 2012; Windig et al. 2012). Some authors, as Haberland et al. (2014), suggested favoring methods that measure compound concentration over sensory methods for genetic selection, because androstenone and skatole have high heritability estimates. However, sensory analysis can reflect consumer acceptance, while chemical analysis has to rely on thresholds to classify a carcass as tainted or not. In addition, the laboratory and sample preparation protocols used can influence the results of chemical analysis (Ampuero Kragten et al. 2011; Haugen et al. 2012). The interpretation of results from chemical analysis uses concentration in fat. However, the percentage of adipose tissue may vary among animals.

A further factor is the establishment of thresholds for compounds’ concentration in chemical analysis. A threshold of 1.0 μg/g for androstenone (Desmoulin et al. 1982; Walstra et al. 1999) and of 0.25 μg/g for skatole (Xue et al. 1996) has frequently been suggested. Meanwhile, lower levels, 0.5 μg/g for androstenone and 0.026 μg/g for skatole, have also been suggested (Annor-Frempong et al. 1997; Aldal et al. 2005). Mathur et al. (2012) observed that the use of the threshold of 1 μg/g and 0.25 μg/g for androstenone and skatole, respectively, resulted in a substantial rejection of carcasses that may not be tainted. Some studies have shown that higher thresholds for androstenone (2–3 μg/g) can be considered when skatole is present in lower levels (< 0.1 μg/g) (Bonneau and Chevillon 2012; Mörlein et al. 2016). As shown by Mörlein et al. (2016), not only the concentration of androstenone, skatole, and indole but also the interactions among them can influence the perception of odor. This can be another reason for some discrepant results between sensory and chemical analysis (Aluwé et al. 2012). Moreover, other compounds, as commented above, which are not considered in the usual chemical analysis of androstenone, skatole, and indole, can be involved in odor perception. Finally, consumers’ sensibility is variable and depends upon age, gender, and country (Dijksterhuis et al. 2000; Matthews et al. 2000; Font-i-Furnols 2012); therefore HNS will provide only subjective scores. For all these reasons and given the specific nature of the different boar taint traits, a multitrait approach combining different phenotypes and correlated traits might be the best choice as all methods have limitations. Even though, it is unclear how to weight each trait in the model, an aspect still needs extra discussion.

## Factors influencing boar taint compounds

Prevalence of boar taint depends upon several factors, such as the raising environment, diet, and breed. Here we will focus on some of these factors that could affect incidence of boar taint for which the interaction with the host genome might play an important role.

As skatole and indole are produced by bacteria in the gut, their availability depends upon the levels of tryptophan, bacterial activity, the presence of specialized bacteria, and the absorption rate in the intestine (Wesoly and Weiler 2012). The influence of the intestinal microbiota composition on boar taint has been investigated.

The levels of pathogenic bacteria (Okrouhlá et al. 2020) and *Clostridium perfringens* (Vhile et al. 2012), a genus reported as skatole producers, were decreased in non-castrated males fed with Jerusalem artichoke, a fermentable fiber source, which resulted in a decrease in skatole levels in the hindgut and fat tissue. Another fermentable fiber source, chicory root, showed a skatole-reducing effect (Li et al. 2019). However, this result did not coincide with a reduced number of bacterial species reported as skatole producers. Nonetheless, the number of *Olsenella scatoligenes* bacteria increased in animals fed with chicory root. These studies showed that an effect of interaction of diet × microbiota × boar taint is possible. Another factor that should be investigated is the interaction of microbiome composition and the host genome as recent studies have shown an effect of interaction between intestinal microbiome and the host genome in other complex traits (Camarinha-Silva et al. 2017; Difford et al. 2018; Maltecca et al. 2020). The microbiome composition and its interaction with the host genome could help to elucidated differences in animals with high and low levels of boar taint.

Boar taint prevalence is variable among breeds, probably due to different selection goals each breed has historically undergone. Higher levels of androstenone levels were observed in fat tissue of Duroc when compared to Landrace boars (Xue et al. 1996; Tajet et al. 2006; Grindflek et al. 2011). Aluwé et al. (2011b) observed higher skatole and androstenone levels in Large White than Pietrain boars. These authors also observed higher levels of boar odor in Large White compared to Pietrain pigs using sensory analysis.

Overall, higher levels of boar taint compounds are observed in dam compared to sire lines (Knol et al. 2010; Windig et al. 2012; Mathur et al. 2013). This is probably due to the correlation between boar taint and other economically important traits. Dam lines are selected, mainly for reproduction traits, whereas sire lines are mainly selected for production traits. As androstenone is synthetized along with other sexual hormones (Grindflek et al. 2011), selection for reproduction traits could have caused boar taint increase in dam lines. This information should be considered in breeding programs, and more attention should be given to dam lines by selecting against boar taint.

However, a conclusion on the influence of breeds or populations on boar taint is hard to formulate. Some factors, such as age, live weight, and management conditions, should be considered as boar taint is influenced by them as well. Also, further studies should be performed by considering the effect of the microbiome and the host genome interaction on boar taint traits.

## Heritability of boar taint traits and genetic correlation with other traits

By focusing on a new trait for selection, heritability estimates as well as genetic correlation with other traits are important parameters that have to be known. Androstenone and skatole deposition have heritability estimates from medium to high magnitudes, i.e., ranging 0.55–0.88 for androstenone and 0.23–0.55 for skatole (Sellier and Bonneau 1988; Tajet et al. 2006; Rowe et al. 2014; Parois et al. 2015), whereas heritability estimates for human nose score ranging 0.11–0.19 (Windig et al. 2012; Mathur et al. 2013). Hence, selection against boar taint is feasible taking heritability into account.

Selection against boar taint may affect other economically important traits in pigs. Therefore the genetic correlation with those traits has to be known. This is the reason why genetic correlations between boar taint and other traits have been investigated intensively (Bergsma et al. 2007; Engelsma et al. 2007; Merks et al. 2010; Mathur et al. 2013).

The main concern involves reproduction traits (Table 1). As androstenone synthesis is stimulated along with other testicular steroids, selection against boar taint could have negative effects in sexual maturity.

**Table 1:** Genetic correlations of reproduction and aggressiveness traits with boar taint compounds and human nose scores (HNS)

Traits	AND	SKA	IND	HNS	Breed	Reference
Age at first insemination	− 0.07	− 0.32	− 0.46	− 0.20	LY	Mathur et al. (2013)
	0.04	0.16	0.27	− 0.10	P	Mathur et al. (2013)
	0.24	0.03	0.12	–	SSL	Engelsma et al. (2007)
Gestation length	0.06	− 0.03	− 0.04	0.18	LY	Mathur et al. (2013)
	− 0.14	0.15	0.07	− 0.06	P	Mathur et al. (2013)
Gilt puberty status	0.22	–	–	–	LW/L	Sellier et al. (2000)
IS	− 0.07	− 0.08	− 0.15	− 0.00	LY	Mathur et al. (2013)
IWI2	− 0.44	0.34	0.29	–	SSL	Engelsma et al. (2007)
Litter mortality	− 0.04	− 0.04	− 0.07	− 0.05	LY	Mathur et al. (2013)
	− 0.09	− 0.09	− 0.10	− 0.03	P	Mathur et al. (2013)
	− 0.59	− 0.35	–	–	SSL	Engelsma et al. (2007)
LP5	− 0.18	0.05	–	–	DL	Strathe et al. (2013)
Number stillborn	− 0.24	0.32	0.13	− 0.36	P	Mathur et al. (2013)
	0.04	0.07	− 0.11	0	LY	Mathur et al. (2013)
Parity at culling	− 0.27	− 0.24	− 0.3	− 0.03	LY	Mathur et al. (2013)
PI	− 0.08	0.20	− 0.11	0.41	LY	Mathur et al. (2013)
	0.03	0.41	0.17	− 0.17	P	Mathur et al. (2013)
Total number born	− 0.22	0.12	− 0.07	0.01	LY	Mathur et al. (2013)
	0.21	− 0.07	− 0.05	0.11	P	Mathur et al. (2013)
	− 0.06	0.16	0.08	–	SSL	Engelsma et al. (2007)
	− 0.12	0.06	–	–	DL	Strathe et al. (2013)
GBU	0.45	0.35	0.48	–	NL	Tajet et al. (2006)
	0.38	0.57	0.53	–	Duroc	Tajet et al. (2006)
	0.65	–	–	–	LW/L	Sellier et al. (2000)
Testes weight	0.47	–	–	–	LW/L	Sellier et al. (2000)
Av. sperm cells^1^	− 0.08	0.03	–	–	C3	Merks et al. (2010)
Concentration^1^	− 0.02	− 0.08	–	–	C3	Merks et al. (2010)
Motility after 1 day^1^	− 0.04	− 0.18	–	–	C3	Merks et al. (2010)
Motility in first^1^	0.03	− 0.13	–	–	C3	Merks et al. (2010)
Primary defects^1^	0.06	− 0.09	–	–	C3	Merks et al. (2010)
Secondary defects^1^	− 0.08	− 0.07	–	–	C3	Merks et al. (2010)
Volume^1^	− 0.04	0.09	–	–	C3	Merks et al. (2010)
LESC	− 0.06	0.05	− 0.30	–	P	Parois et al. (2015)
	− 0.17	0.03	0.007	–	PLW	Parois et al. (2015)
	0.15	–	–	–	P	Dugué et al. (2020)
	− 0.26	–	–	–	PLW	Dugué et al. (2020)
LESFE	0.24	–	–	–	P	Dugué et al. (2020)
	0.89	–	–	–	PLW	Dugué et al. (2020)
LESBS	0.21	–	–	–	P	Dugué et al. (2020)
	0.1	–	–	–	PLW	Dugué et al. (2020)

^1^Correlations between estimated breeding values for boar taint compounds and reproduction traits; *IS* Inseminated for 2nd parity, *IWI2* interval weaning 2nd insemination, *GBU Glandula bulbourethralis*, *PI* prolonged rebreeding interval, *LP5* live piglets at d 5, *LESC* lesions on carcass, *LESFE* lesions at fattening stage entrance, *LESBS* lesions before slaughter, *LY* Landrace and Yorkshire, *NL* Norwegian Landrace, *LW/L* line with Large White × Landrace background, *SSL* synthetic sire line, *C3* crosses among lines with Pietrain, Large White, and Duroc background, *DL* Danish Landrace, *P* Pietrain, *PLW* Pietrain × Large White

Age at first insemination showed a favorable correlation with androstenone in sire (Engelsma et al. 2007; Mathur et al. 2013), but an unfavorable correlation in dam lines (Mathur et al. 2013). Androstenone has been exhibited to be negatively correlated with total number of piglets born (Engelsma et al. 2007; Mathur et al. 2013; Strathe et al. 2013), and litter mortality (Engelsma et al. 2007; Mathur et al. 2013) as well. Thus, decreasing androstenone should increase the number of piglets born, but it will increase mortality. These same authors observed an unfavorable correlation between total number of piglets born and litter mortality, and skatole, indole, and HNS. Engelsma et al. (2007) reported a negative correlation between androstenone and interval weaning 2nd insemination, while Mathur et al. (2013) observed a negative correlation between boar taint compounds and insemination for second parity. Therefore, these studies have shown that selecting against boar taint could have negative effects on some reproduction traits.

Nevertheless, few studies have been investigating the boar taint correlation with male fertility traits. Merks et al. (2010) evaluated the correlation between several estimated breeding values (EBVs). These authors observed low and negative correlation values between androstenone and volume, concentration, and motility of sperm. Bergsma et al. (2007) also reported low genetic correlations, but positive correlation between androstenone and volume, motility, and longevity of the semen, whereas negative values were found between skatole and the traits of motility and longevity. Other studies, aiming to evaluate correlation effects on boars’ maturity, have used the development of the glandula bulbourethralis*.* These studies reported an unfavorable correlation with boar taint compounds (Sellier et al. 2000; Tajet et al. 2006). Thus, selection against boar taint could retard boars’ maturity.

Despite the unfavorable correlation with reproduction traits, favorable correlations have been reported with production traits (Table 2). Boar taint has shown to have a favorable correlation with backfat and meat percentage (Sellier et al. 2000; Engelsma et al. 2007; Merks et al. 2010; Windig et al. 2012; Haberland et al. 2014; Dugué et al. 2020). A favorable correlation was also reported between feed conversion rate (FCR) and androstenone and skatole (Strathe et al. 2013; Haberland et al. 2014; Dugué et al. 2020). Nevertheless, Strathe et al. (2013) reported a negative correlation between FCR and androstenone. Average daily gain (ADG) has been presented to have an unfavorable correlation with androstenone (Sellier et al. 2000; Strathe et al. 2013; Haberland et al. 2014), and a favorable correlation with skatole (Windig et al. 2012; Strathe et al. 2013; Haberland et al. 2014). Despite the unfavorable effect on growth rate, decreasing androstenone and skatole would improve feed conversion. In addition, we can expect that selecting against these two compounds would increase lean meat and decreasing fat depth in the carcass.

**Table 2:** Genetic correlations of production traits with boar taint compounds and human nose scores (HNS)

Traits	AND	SKA	IND	HNS	Breed	Reference
ADG	− 0.06	− 0.1	− 0.02	− 0.07	PB	Windig et al. (2012)
	− 0.11	0.07	0.08	–	SL	Merks et al. (2009)
	0.19	− 0.05	0.06	–	CL	Haberland et al. (2014)
	0.1	− 0.04	–	–	DL	Strathe et al. (2013)
	0.19	− 0.33	–	–	C3	Merks et al. (2010)
	0.04	–	–	–	LW/L	Sellier et al. (2000)
	− 0.16	–	–	–	P	Dugué et al. (2020)
	0.16	–	–	–	PLW	Dugué et al. (2020)
Backfat	0.17	0.12	0.15	0.29	PB	Windig et al. (2012)
	0.2	0.17	0.27	–	SSL	Engelsma et al. (2007)
	0.26	− 0.01	–	–	C3	Merks et al. (2010)
	0.21	− 0.07	− 0.14	–	SL	Merks et al. (2009)
	0.27	0.01	0.15	–	CL	Haberland et al. (2014)
	0.11	–	–	–	LW/L	Sellier et al. (2000)
Ultrasonic backfat	0	0.07	0.13	–	SSL	Engelsma et al. (2007)
Drip loss	− 0.05	0.06	− 0.1	–	CL	Haberland et al. (2014)
	0.09	0.11	–	–	C3	Merks et al. (2010)
	0.08	–	–	–	P	Dugué et al. (2020)
	0.4	–	–	–	PLW	Dugué et al. (2020)
FCR	0.13	0.14	0.16	–	CL	Haberland et al. (2014)
	− 0.04	0.18	–	–	DL	Strathe et al. (2013)
	0.47	–	–	–	P	Dugué et al. (2020)
	0.51	–	–	–	PLW	Dugué et al. (2020)
pH L	− 0.20	–	–	–	P	Dugué et al. (2020)
	− 0.10	–	–	–	PLW	Dugué et al. (2020)
pH H	− 0.40	–	–	–	P	Dugué et al. (2020)
	− 0.23	–	–	–	PLW	Dugué et al. (2020)
IMF	0.19	− 0.04	0.14	–	CL	Haberland et al. (2014)
	− 0.04	–	–	–	P	Dugué et al. (2020)
	0.32	–	–	–	PLW	Dugué et al. (2020)
Loin depth	0.21	− 0.03	− 0.03	–	SL	Merks et al. (2009)
	− 0.01	− 0.11	0.09	–	SSL	Engelsma et al. (2007)
JC	− 0.05	0.42	–	–	C3	Merks et al. (2010)
Lean meat	− 0.2	− 0.19	− 0.22	–	SSL	Engelsma et al. (2007)
	− 0.22	− 0.12	− 0.21	–	CL	Haberland et al. (2014)
	− 0.18	− 0.2	–	–	DL	Strathe et al. (2013)
	− 0.26	–	–	–	P	Dugué et al. (2020)
	− 0.37	–	–	–	PLW	Dugué et al. (2020)
Live growth	0.34	0.18	0.35	–	SSL	Engelsma et al. (2007)
LM	0.04	0.15	–	–	C3	Merks et al. (2010)
Meat surface	− 0.23	− 0.16	− 0.2	–	CL	Haberland et al. (2014)

*ADG* average daily gain, *FCR* feed conversion ratio, *pH L* pH in longissimus dorsi, *pH H* pH in ham, *IMF* intramuscular fat, *JC* Japanese color scale, *LM* loin marbling score, *LW/L* line with Large White × Landrace background, *SSL* synthetic sire line, *SL* synthetic line derived from Duroc, Landrace, Yorkshire, and Pietrain, *C3* crosses among lines with Pietrain, Large White, and Duroc background, *CL* commercial line, *PB* data of crosses between sire and dam lines and purebred with Duroc, Large White, Pietrain, Landrace, and Yorkshire background, *DL* Danish Landrace, *P* Pietrain, *PLW* Pietrain × Large White

Intramuscular fat is also expected to decrease, once that an unfavorable correlation was reported between intramuscular fat and androstenone and indole (Haberland et al. 2014; Dugué et al. 2020). However, drip loss and meat pH have shown a favorable correlation with androstenone (Merks et al. 2010; Dugué et al. 2020). More studies considering genetic correlation between boar taint and meat quality traits must be performed.

The relation between boar taint and fatty acid composition is also related to meat quality but not well studied so far. Mörlein and Tholen (2015) evaluated fatty acid composition in entire male pigs with divergent levels of boar taint compounds. These authors found that saturated fatty acids (SFA) increased in entire males with high levels of androstenone and skatole, while polyunsaturated fatty acids (PUFA) increased in boars with low levels of androstenone and skatole. On the other hand, Liu et al. (2017) found that PUFA were positively correlated with androstenone. These authors also have shown that monounsaturated fatty acids (MUFA) content was negatively correlated with androstenone and skatole, and no difference was found regarding SFA. In addition, Verplanken et al. (2017) found higher MUFA content in tainted animals compared to untainted animals. Other studies in which fatty acid composition was compared among entire males, castrated and immunocastrated have been performed. The results have shown lower abundance of SFA and higher abundance of PUFA in entire males compared to castrated and immunocastrated animals (Pauly et al. 2012; Mackay et al. 2013; Zoels et al. 2020). These studies showed that raising entire males and selecting against boar taint compounds can affect the fatty acid composition of adipose tissue.

The other aspect that concerns farmers regarding raising entire males is aggressiveness. Parois et al. (2015) and Dugué et al. (2020) found low genetic correlation between skin lesions in carcass and androstenone, skatole, and indole. Nevertheless, Dugué et al. (2020) found a high and favorable genetic correlation between androstenone and skin lesions shortly after entering the fattening pen. Androstenone production is related to other steroid hormones, as testosterone (Robic et al. 2008), which is considered to be associated with an aggressive behavior (Prunier et al. 2013). Therefore, selecting against boar taint can have a beneficial response on aggressive behavior. However, aggressiveness (and social behavior in general) is complex to measure since it involves environment conditions and interactions among animals. Additionally, it was shown that dominant pigs have greater concentrations of androstenone, which indicates an important role of androstenone in social interactions (Parois et al. 2017). Thus, further studies focusing on how selection against androstenone can affect aggressiveness and social interactions are needed.

It is worth to mention that most correlation values reported in those studies were low; thus, selecting against boar taint could have less pronounced effects on other traits. As boar taint is not yet widely considered in breeding programs, the realized response to selection on boar taint through correlated traits is not yet clear. Likewise, how much time it would take to reduce the incidence of males with boar taint is unclear. In addition, further studies are required in order to investigate the effects of raising entire males and selection against boar taint on the fatty acid composition in fat tissue and, consequently, on the meat quality. Thus, in the next section, we will discuss some selection studies presented in the literature.

## Selecting against boar taint

Haberland et al. (2014) evaluated different breeding scenarios. A first reference scenario did not have the selection against boar taint in its breeding goals, whereas the two other scenarios did take selection against boar taint into account. One scenario used chemical analysis of androstenone, skatole, and indole, and the other used HNS. The authors observed a decrease in boar taint even when there was no selection against this trait, due to a favorable correlation between boar taint reduction and other traits, such as lean meat percentage. The best results, in terms of genetic gains and costs per selection candidate, were observed by using the breeding scheme with selection against androstenone, skatole, and indole in the breeding goals. However, even though the economic gain was higher, the cost of analyses was also high. Nonetheless, these authors stated that this breeding scenario could be a worthy alternative. Regarding the time to reduce boar taint, these authors demonstrated that androstenone, skatole, and indole could be reduced by 50% in 7, 6, and 8 years of selection, respectively. In addition, androstenone could be reduced to levels below a threshold of 0.5 μg/g in 4 years. Furthermore, they observed a favorable response in lean meat and feed conversion rate but negative effects in drip loss, ADG, and intramuscular fat. These results suggest therefore also that caution is needed by selecting against boar taint.

On the other hand, a simulation study by Merks et al. (2009) demonstrated that a combined index could be used for the reduction of boar taint while keeping genetic progress in the production traits. In this study, three scenarios were also considered: selection only for production traits; selection only for boar taint; and a combined selection. These authors reported that it would take 4 years of selection against boar taint to reduce androstenone and skatole concentrations below the suggested thresholds (< 2.56 μg/g for androstenone and < 0.20 μg/g for skatole).

Regarding other traits, Mathur et al. (2013) have compared a selection index composed only by reproduction traits and a combined selection index. They observed a minor loss in dam lines regarding the total number of piglets born when boar taint was included in the selection indexes. These authors stated that this happened due to the decrease in pressure on this trait rather than an unfavorable correlated response. They also reported higher economic gains by using a combined index when there is an incentive for intact males by the market. On this subject, Backus et al. (2016) reported the need of better payments by the market; otherwise, it would not be profitable to change the aim for selection against boar taint and to preventively change the environmental conditions at farms.

In summary, these studies showed that the best option is to use a combined selection index which includes boar taint. The key point is to choose the most appropriate economic weight for each trait. In addition, in order to reduce boar taint, more emphasis might be needed towards dam lines, in which higher levels of boar taint compounds were reported.

## Candidate genes

QTL studies and gene expression analyses can help to find possible genes affecting boar taint. Several studies have been conducted on searching for those genes; results from these studies are summarized in Tables 3 and 4.

**Table 3:** Chromosomes where QTLs associated with boar taint have been found

Chromosome	Trait	Reference
1	Androstenone	Duijvesteijn et al. (2010)
2	Androstenone	Lee et al. (2005)
	Indole	Bidanel et al. (2006)
3	Androstenone	Quintanilla et al. (2003)
4	Intensity of smell and taste	Grindflek et al. (2001)
	Androstenone	Lee et al. (2005)
	Androstenone	Quintanilla et al. (2003)
6	Indole	Bidanel et al. (2006)
	Androstenone	Duijvesteijn et al. (2010)
	Androstenone	Duijvesteijn et al. (2014)
	Intensity of smell and taste	Grindflek et al. (2001)
	Androstenone	Grindflek et al. (2011)
	Androstenone	Lee et al. (2005)
	Androstenone	Quintanilla et al. (2003)
	Skatole	Ramos et al. (2011)
	Skatole	Varona et al. (2005)
7	Skatole and indole	Bidanel et al. (2006)
	Intensity of smell and taste	Grindflek et al. (2001)
	Androstenone	Lee et al. (2005)
	Androstenone	Milan et al. (1998)
	Androstenone	Quintanilla et al. (2003)
9	Androstenone	Lee et al. (2005)
	Androstenone	Quintanilla et al. (2003)
12	Skatole	Bidanel et al. (2006)
14	Skatole, indole, and pork odor	Lee et al. (2005)
	Androstenone	Quintanilla et al. (2003)
X	Skatole	Bidanel et al. (2006)

**Table 4:** Results of differential gene expression studies associated with boar taint in the liver and testis tissue

Tissue	Gene	Expression status	Reference
Liver	*CYP2E1*, *CYP2A19*	Downregulated in high androstenone	Moe et al. (2008)
	*3βHSDs*, *17βHSDs*	Upregulated in low androstenone	Doran et al. (2004); Nicolau-Solano et al. (2006); Chen et al. (2007)
	*HSD17B2*	Downregulated in high androstenone and skatole	Moe et al. (2008); Gunawan et al. (2013a, b)
	*AKR1D1*	Downregulated in high androstenone	Moe et al. (2008)
Testis	*CYP11A1*, *CYP2C33*, *CYP17A1*	Upregulated in high androstenone	Grindflek et al. (2010); Gunawan et al. (2013b); Leung et al. (2010); Moe et al. (2007b)
	*CYB5*	Upregulated in high androstenone	Grindflek et al. (2010); Leung et al. (2010); Moe et al. (2007b)
	*CYB5*	Upregulated in high boar taint	Drag et al. (2017)
	*HSD17B4*	Upregulated in high androstenone	Moe et al. (2007b); Grindflek et al. 2010; Leung et al. (2010)
	*AKR1C2/3/4*	Upregulated in high androstenone	Moe et al. (2007b); Grindflek et al. (2010); Leung et al. (2010)
	*SULT2A1*, *SULT2B1*	Upregulated in high androstenone	Moe et al. (2007b); Grindflek et al. (2010); Leung et al. (2010)

Briefly, we know that *CYP11A1*, *CYP17A1*, and *CYB5* genes are involved in the first steps of androstenone production, as they are responsible for steroid synthesis (Davis and Squires 1999; Lin et al. 2005; Moe et al. 2007b). In the same way, genes from the hydroxysteroid dehydrogenase family, such as *HSD17B4*, have a role in androstenone synthesis in the testis (Payne and Hales 2004). However, other genes from this same family, such as *3βHSD* e *17βHSD*, seem to be involved in androstenone degradation in the liver (Doran et al. 2004; Nicolau-Solano et al. 2006; Chen et al. 2007). The genes *SULT2A1* and *SULT2B1*, responsible for androstenone sulfoconjugation, are also involved with androstenone degradation (Sinclair et al. 2005a, b). Although these genes are upregulated in high androstenone animals (Table 4), lower levels of testicular and hepatic *SULT2A1* and *SULT2B1* protein have been related to high levels of androstenone in fat (Sinclair et al. 2006; Moe et al. 2007a; Drag et al. 2017). These divergent results may be due to different mechanism of controls over the mRNA transcript and protein of these genes. The genes *CYP2A6* and *CYP2E1*, located in QTL regions for boar taint (Lee et al. 2005; Varona et al. 2005; Duijvesteijn et al. 2010), are related to skatole degradation and seem to be inhibited by androstenone (Doran et al. 2002; Chen et al. 2008).

Several other regions have been related to this trait, showing its polygenic nature. As suggested by Große-Brinkhaus et al. (2015), the polygenic inheritance of boar taint makes this trait a good candidate for genomic selection. Here, we will focus on most recently found candidate genes.

Genes from the family glutathione S-transferases (*GSTO1*, *MGST1*) are most known for the catalyzation of conjugation reactions of fatty acids, xenobiotics, and products of oxidative processes (Moe et al. 2008). These genes were downregulated in liver tissue of high boar taint animals (Moe et al. 2008; Drag et al. 2017, 2018) and upregulated in the testis of high boar taint animals (Moe et al. 2007b; Leung et al. 2010). However, Gunawan et al. (2013a) reported *GSTO2* and *GSTM2* to be upregulated in the testis of high skatole animals.

The steroidogenic acute regulatory protein (*STAR*) has been found to be upregulated in the testis of animals with high boar taint (Moe et al. 2007b; Grindflek et al. 2010; Leung et al. 2010; Drag et al. 2017). The *STAR* gene regulates the transport of cholesterol from outer to inner mitochondrial membrane, where it is converted to pregnenolone (Christenson and Strauss 2000). Sahadevan et al. (2015), working with gene expression in liver tissues of animals with low and high androstenone, have generated gene co-expression clusters. In these clusters, genes previously related to boar taint (HSD family) and genes from the solute carrier (SLC), UDP glucuronosyltransferase (UGT), and aldehyde dehydrogenase (ALDH) families were reported. Drag et al. (2019) found eQTLs associated with *CYP1A2* and *CYB5D1*, previously reported for boar taint and new candidates, leucine rich repeat and fibronectin type III domain containing 2 (*LRFN2*), glutamate ionotropic receptor kainate type subunit 1 (*GRIK1*), ras association domain family member 4 (*RASSF4*), and sphingosine kinase 2 (*SPHK2*), associated with low androstenone.

Focusing on gene pathways, Sahadevan et al. (2014) constructed an interaction network based on whole gene expression between samples with divergent androstenone levels in the testes. Aiming to identify significant interactions among genes and to relate them with key pathways for androstenone synthesis, these authors reported 718 significant genes enriched in 92 pathways. Among these pathways, the authors highlighted the steroid hormone synthesis, the glutathione metabolism, sphingolipid metabolism, fatty acid metabolism, and cyclic AMP—PKA/PKC signaling. In these pathways, genes from cytochrome P450, HSD, and glutathione S-transferases families, which were previously related to boar taint, were found to have a significant interaction among them. In addition, *SPHK2* and genes from the UGT family, also found in recent studies (Sahadevan et al. 2015; Drag et al. 2019), were found to have a significant interaction.

In recent years, epigenetic studies are emerging that potentially help us understand the mechanisms behind these differences in gene expression and gene pathways. Differences in the epigenome can also have effects on boar taint. The methylation profile of the testis from animals with boar taint has been analyzed (Wang and Kadarmideen 2019a, b). Wang and Kadarmideen (2019b) have found differences in the methylation site of genes previously associated with boar taint (*ACACA*, *CYP21A2*, *CYP27A1*, *HSD17B2*, *LHB*, *PARVG*, and *SERPINC1*). Wang and Kadarmideen (2019a) showed that epigenomes change between animals with high and low boar taint. The authors found differentially methylated CpG sites associated with boar taint in the genes *CRYL1*, *DNMT3A*, *EGFR*, *FASN*, *PEMT*. Fatty acid synthase (*FASN*) plays a central role in the fatty acid biosynthesis pathway that is correlated with boar taint compounds (Sahadevan et al. 2014; Liu et al. 2017). DNA methyltransferase 3 alpha (*DNMT3A*) encodes an enzyme responsible for DNA methylation. Epidermal growth factor receptor (*EGFR*) and phosphatidylethanolamine N-methyltransferase (*PEMT*), although not well elucidated, might be involved in estrogen regulation.

Another way to use genetics to reduce boar taint can lay in the use of genome editing approach, as CRISPR/Cas. The *SRY* gene (sex-determining region), located on the Y chromosome and involved in male sexual development, can be targeted. This gene is subject of studies which use genome editing tools in order to control the sexual development by suppressing the male gender development in embryogenesis, which results in female phenotype (Kurtz and Petersen 2019). Without a male phenotype, boar taint is no longer an issue. Other genes potentially involved in sexual differentiation, but located on autosomal chromosomes, can also be a target, such as *SOX9* and *KISSR* (Sonstegard et al. 2017; Stachowiak et al. 2017). However, the social acceptance of CRISPR/Cas is still a controversial issue.

New genes have been reported along with important pathways and new mechanisms of gene expression control. These studies have brought us closer to understand the synthesis and metabolism of androstenone and skatole. Also, these results could help to choose candidate genes to be used in a breeding system aiming to reduce boar taint. Genes involved in degradation of boar taint compounds, such as *SULT2A1* and *SULT2B1*, seems to be a good option to focus.

## Genetic and genomic evaluation

Regarding the methods used to evaluate genetically boar taint, several interesting points have been presented. Some authors, as Haberland et al. (2014), suggested that it is better to use compounds concentration to select animals due to their high heritability. On the other hand, Windig et al. (2012) reported that using information of sensory analysis of more relatives may result in similar results obtained by using compounds concentrations. Given the specific nature of the different boar taint traits, multitrait models that would allow grouping different boar taint traits but also allow including other traits have therefore been suggested (Strathe et al. 2013). As for other difficult to measure traits, using genome-assisted breeding value prediction would be an interesting option (de Campos et al. 2015). Different types of genomic evaluation models could be qualified, but most likely multitrait single-step models allowing the inclusion of information obtained in GWAS studies, adapted for crossbreeding, should be considered and studied (Alvarenga et al. 2020; Misztal et al. 2020).

Therefore, the genetic and genomic evaluation of boar taint will need careful choices of traits, models, and methods, taking into account reported issues in order to allow performing a successful selection against boar taint. It should be clarified that recording boar taint on the slaughter line will remain as a routine process in an entire males raising system, once it is not possible to decrease the risk of boar taint to zero. However, this would also allow permanent phenotyping and detection of tainted meat. The proposed multitrait methods could then combine this data with other less abundant traits, all this recorded across different animals including close relatives of breeding animals (full-brothers). Also, genomic information could be added to detect boars and boar lines that show higher risks of tainted meat.

## Conclusion

Animal welfare concerns are strongly increasing in animal production. Currently, in pig production, important concerns are related to aggressiveness, penile biting, and need for surgical castration. Surgical castration still exists, as it is the easiest practice to prevent boar taint meat. However, due to animal welfare concerns, castration is currently in the process to be banished. Boar taint depends upon factors such as breed and therefore the genetics of the animals, age, diet, and raising conditions. It was also shown that an effect of interaction of diet × microbiota × host genome is possible and should be further investigated. In addition to changes in diet and environment conditions, genetic selection should be considered as an important option to reduce boar taint. Management changes may also require adaptations of breeding goals. For example, the aggressiveness might become an issue to consider when raising entire males. Besides optimal methods for grouping animals, breeding for less aggressivity might be required.

Genetic selection seems to be the most feasible and cost-effective alternative to surgical castration, since androstenone and skatole have a high heritability. However, as already put forward, the response to selection on other traits should also be considered. To select against boar taint may bring losses on reproduction and gain on production traits. The use of combined selection indexes seems to be the best alternative to ensure improvements on boar taint without jeopardizing other traits. Multitrait models grouping different boar taint traits and phenotypic information on different types of animals, including close relatives of breeding animals (full-brothers), should be considered as boar taint is a complex and difficult to assess condition.

Genes and QTLs related to boar taint have been identified. Some genes had their roles described, while others have not a well determined function yet. This review shows the current status in the very dynamic evolution of research in the fields of genetics of boar taint reduction as an alternative to castration. Ongoing studies are searching for other genes involved in boar taint. Knowledge of these genes might allow direct action on them by breeding or through using biotechnological tools such as CRISPR/Cas.

## Data Availability

Not applicable.
